# A Small Molecule Inhibitor of Pex3–Pex19 Interaction Disrupts Glycosome Biogenesis and Causes Lethality in *Trypanosoma brucei*

**DOI:** 10.3389/fcell.2021.703603

**Published:** 2021-07-19

**Authors:** Hiren Banerjee, Paul LaPointe, Gary Eitzen, Richard A. Rachubinski

**Affiliations:** Department of Cell Biology, University of Alberta, Edmonton, AB, Canada

**Keywords:** neglected tropical disease, trypanosome, glycosome, organelle biogenesis, protein–protein interaction, yeast two-hybrid, drug screening, small molecule inhibitor

## Abstract

Trypanosomatid parasites, including *Trypanosoma* and *Leishmania*, are infectious zoonotic agents for a number of severe diseases such as African sleeping sickness and American trypanosomiasis (Chagas disease) that affect millions of people, mostly in the emergent world. The glycosome is a specialized member of the peroxisome family of organelles found in trypanosomatids. These organelles compartmentalize essential enzymes of the glycolytic pathway, making them a prime target for drugs that can kill these organisms by interfering with either their biochemical functions or their formation. Glycosome biogenesis, like peroxisome biogenesis, is controlled by a group of proteins called peroxins (Pex). Pex3 is an early acting peroxin that docks Pex19, the receptor for peroxisomal membrane proteins, to initiate biogenesis of peroxisomes from the endoplasmic reticulum. Identification of Pex3 as the essential master regulator of glycosome biogenesis has implications in developing small molecule inhibitors that can impede Pex3–Pex19 interaction. Low amino acid sequence conservation between trypanosomatid Pex3 and human Pex3 (HsPex3) would aid in the identification of small molecule inhibitors that selectively interfere with the trypanosomatid Pex3–Pex19 interaction. We tested a library of pharmacologically active compounds in a modified yeast two-hybrid assay and identified a compound that preferentially inhibited the interaction of *Trypanosoma brucei* Pex3 and Pex19 versus HsPex3 and Pex19. Addition of this compound to either the insect or bloodstream form of *T. brucei* disrupted glycosome biogenesis, leading to mislocalization of glycosomal enzymes to the cytosol and lethality for the parasite. Our results show that preferential disruption of trypanosomal Pex3 function by small molecule inhibitors could help in the accelerated development of drugs for the treatment of trypanosomiases.

## Introduction

Neglected tropical diseases (NTDs) are a group of infectious parasitic diseases that threaten the populations of many emerging nations (Stuart et al., 2008; Mitra and Mawson, 2017; World Health Organization, 2017). Sleeping sickness is a NTD of sub-Saharan Africa whose infectious agent is the protozoan parasite, *T. brucei*. Left untreated, sleeping sickness is fatal. Current drugs such as melarsoprol, suramin, pentamidine, eflornithine, fexinidazole, and nifurtimox have shown efficacy in treating sleeping sickness, but their utility can be restricted because of toxicity, severe side effects, and complicated administration (Nwaka and Hudson, 2006; Giordani et al., 2016; de Rycker et al., 2018; De Koning, 2020). Moreover, NTDs impact mostly those countries that lack the financial or infrastructural resources needed to develop or deliver new therapies. Therefore, the identification of novel drug targets and the development of new drugs for these targets remain ongoing pursuits.

Trypanosomatid parasites contain a specialized peroxisome called the glycosome. Glycosomes compartmentalize enzymes of the glycolytic pathway, which are located in the cytosol of cells of other organisms (Guerra-Giraldez et al., 2002; Haanstra et al., 2008, 2016). Because glycosomes are both unique to trypanosomatids and house essential metabolic enzymes (Haanstra et al., 2016; Crowe and Morris, 2021), they are an ideal target for drug development. The biogenesis of glycosomes is controlled by *PEX* genes that encode proteins called peroxins (Pex). Pex3 docks Pex19, the receptor for peroxisomal membrane proteins (PMPs), which results in the biogenesis of peroxisome precursors from the endoplasmic reticulum (ER) (Ghaedi et al., 2000; Fang et al., 2004; Smith and Aitchison, 2013). We identified the long sought trypanosomal Pex3 (TbPex3) by an analysis of human Pex3 (HsPex3) through the HHpred bioinformatics platform, which looks for similarities in protein secondary structure rather than for similarities in protein primary structure (Banerjee et al., 2019). TbPex3 was also identified independently using biochemical means (Kalel et al., 2019). Reduction in the amounts of TbPex3 led to reduced numbers of enlarged glycosomes and to the mislocalization of glycosomal matrix enzymes to the cytosol in both procyclic form (PCF) cells and bloodstream form (BSF) cells of *T. brucei*. Notably, reductions in the amount of TbPex3 led to death of all PCF and BSF cells.

Trypanosomal Pex3 exhibits only 7% amino acid identity with HsPex3 but does maintain a relatively conserved Pex19 interaction domain (Banerjee et al., 2019; Kalel et al., 2019). This limited primary sequence similarity between TbPex3 and mammalian Pex3 proteins makes TbPex3 and its interactions with protein partners attractive therapeutic targets. We used the yeast two-hybrid assay to reconstitute the trypanosomal and HsPex3–Pex19 interaction in *Saccharomyces cerevisiae*. We then screened a library of pharmacologically active compounds (LOPAC 1280) to search for compounds that preferentially inhibit the trypanosomal versus the HsPex3–Pex19 interaction. The screen identified six potential candidate molecules meeting this criterion. Further analyses identified one compound more effective at blocking the interaction between TbPex3 and TbPex19 compared to HsPex3 and HsPex19. Administration of this compound led to compromised glycosome biogenesis in *T. brucei* and was lethal for both PCF and BSF cells of *T. brucei* at concentrations that have limited effect on the viability of human cells.

## Materials and Methods

### Yeast Two-Hybrid Assay

PCR products encoding full-length TbPex3 and full-length HsPex3 were cloned in-frame and downstream of the DNA-binding domain (BD) of the *GAL4* transcriptional activator in pGBT9 (Clontech). Full-length TbPex19 and full-length HsPex19 were cloned in-frame and downstream of the activation domain (AD) of the *GAL4* transcriptional activator in pGAD424 (Clontech). The *S. cerevisiae* strain HF7c deleted for the *PDR5* gene (HF7c *pdr5*Δ) encoding the major drug efflux pump of yeast (Golin and Ambudkar, 2015) was transformed with plasmids, and transformed cells were grown on synthetic dropout medium agar lacking leucine and tryptophan (-Leu -Trp) to determine total cell growth and on synthetic dropout medium agar lacking histidine, leucine, and tryptophan (-His -Leu -Trp) to determine growth of cells exhibiting protein–protein interaction between the AD-fusion and BD-fusion constructs.

### Yeast Two-Hybrid Screening of a Library of Small Molecules for Inhibitors of TbPex3/TbPex19 Interaction

Yeast strain H7Fc *pdr5*Δ expressing BD-TbPex3/AD-TbPex19 or BD-HsPex3/AD-HsPex19 was cultured in complete synthetic medium lacking histidine to an OD_600_ of 0.1. The 10 mM 3-aminotriazole (3AT) was added to the strain expressing BD-HsPex3/AD-HsPex19 to inhibit basal expression of the *HIS3* gene in this strain and to achieve yeast strains expressing trypanosome or human yeast two-hybrid constructs that would be equally sensitive to exogenously added compounds. 0.1 mL of cultures was dispensed into 384-well Matrix WellMate Microplates (Thermo Fisher Scientific), and 1 μL of compound from the LOPAC 1280 was added to each well to give a compound concentration of 100 μM. One microliter of DMSO was added to each well of the second row of each plate as a negative control (no inhibition), while 100 mM 3AT (final concentration) was added to wells of the 23rd row as a positive control (100% inhibition). The wells of the first and last rows contained water. Plates were incubated at 30°C, and after 24 h growth was determined by measuring the OD_600_ of each well using a Synergy HTX Multi-Mode Microplate Reader (BioTek). For each well, percent growth inhibition was calculated as 100 − 100 × (growth with compound/growth no compound). Wells exhibiting >65% growth inhibition of the BD-TbPex3/AD-TbPex19 strain and <65% growth inhibition of the BD-HsPex3/AD-HsPex19 strain defined compounds were considered as preferentially inhibiting the TbPex3/TbPex19 interaction. To identify compounds that non-selectively inhibited yeast growth, the BD-TbPex3/AD-TbPex19 strain was cultured in complete synthetic medium containing histidine but lacking tryptophan and leucine. Compounds from the LOPAC 1280 library that showed growth inhibition under these conditions were considered as general growth inhibitors.

### Assay for Protein Binding

Binding between TbPex3 and TbPex19 and between HsPex3 and HsPex19 was examined essentially as described (Banerjee et al., 2019). MBP fusions to TbPex3 and HsPex3 were constructed in pMAL-c2 (New England Biolabs). 6 × His fusion to TbPex19 and HsPex19 was constructed in pET-30a (Novagen). Recombinant proteins were expressed in *Escherichia coli* strain BL21 (Invitrogen). His-TbPex19 or His-HsPex19 was immobilized on Ni-NTA Agarose (Qiagen) and incubated with purified MBP-TbPex3 or MBP-HsPex3 proteins at 4°C for 2.5 h under non-denaturing conditions suggested by the manufacturer (Qiagen). Twofold dilutions of the small molecule 2,3-dimethoxy-1,4-naphthoquinone (DMNQ) from 200 to 6 μM was added to the incubation to determine its inhibitory effect on the TbPex3–TbPex19 and the HsPex3–HsPex19 interactions. Immobilized proteins were eluted in sample buffer (50 mM Tris–HCl, pH 6.8, 2% SDS, 5% (vol/vol) glycerol, 0.002% bromophenol blue, 100 mM 2-mercaptoethanol) and subjected to immunoblotting with anti-MBP antibody (New England Biolabs) and anti-His-Tag antibody (Sigma-Aldrich).

### Microscale Thermophoresis Determination of Pex3–Pex19 Binding Affinity

The binding affinity between TbPex3 and TbPex19 and between HsPex3 and HsPex19 was determined by microscale thermophoresis (MST) analysis using a Monolith NT.115 instrument under conditions suggested by the manufacturer (NanoTemper Technologies). His-TbPex19 and His-HsPex19 were labeled with NT-647 fluorescent dye (NanoTemper Technologies). Binding affinity of Pex3–Pex19 pairs was determined by combining increasing concentrations of MBP-Pex3 protein (0–2.5 μM) with 100 nM of NT-647-labeled His-Pex19 in MST buffer (50 mM Tris–HCl, pH 7.4, 150 mM NaCl, 10 mM MgCl_2_, 0.05% Tween-20). To analyze the effect of the presence of DMNQ on the binding affinity between TbPex3 and TbPex19 and between HsPex3 and HsPex19, measurements were made using 100 nM each of NT-647-labeled His-Pex19 and MBP-Pex3 and different concentrations of DMNQ from 3 nM to 100 μM. Data analysis was done using Monolith software (NanoTemper Technologies).

### Trypanocidal Activity of the Small Molecule Compound DMNQ

Bloodstream form cells of *T. brucei* Lister 427 were maintained in HMI-9 medium containing 10% fetal bovine serum (FBS), 10% Serum Plus (Sigma-Aldrich) at 37°C with 5% CO_2_. PCF cells of *T. brucei* Lister 427 were maintained in SDM-79 medium (Invitrogen) containing 10% FBS at 25°C with 5% CO_2_. HEK293T cells were maintained in DMEM medium containing 10% FBS at 37°C with 5% CO_2_.

To measure the trypanocidal activity of the small molecule DMNQ, 50 μL of BSF cells (2 × 10^4^ cells mL^–1^), PCF cells (2 × 10^5^ cells mL^–1^), or HEK293T cells (5 × 10^4^ mL^–1^) were seeded into wells of a 96-well Optical-Bottom Plates (Thermo Fisher Scientific). Fifty microliters of DMSO or DMSO containing the small molecule DMNQ were added to wells to achieve a concentration of DMNQ ranging from 0 to 10 μM. Following incubation of cells at their appropriate temperatures for 24 or 48 h in the presence of DMNQ, 100 μL of CellTiter-Glo reagent (Promega) was added to each well, the plate was incubated at room temperature for 30 min, luminescence was measured, and cell viability was determined according to the manufacturer’s protocol.

### Immunofluorescence Microscopy and Image Analysis

Harvesting, fixation, antibody staining, confocal fluorescence microscopy, image deconvolution and image processing of PCF and BSF cells were performed essentially as described (Banerjee et al., 2019).

## Results

### Yeast Two-Hybrid Screening Identifies a Small Molecule Inhibitor of Trypanosomal Pex3–Pex19 Interaction

The Pex3–Pex19 protein complex initiates peroxisome formation at the ER in a diversity of organisms, including the trypanosomatids. We (Banerjee et al., 2019) and others (Kalel et al., 2019) recently identified a trypanosomal ortholog of Pex3 that interacts with trypanosomal Pex19 to initiate glycosome biogenesis. Glycosomes are specialized peroxisomes that have been shown to be essential for the viability of *T. brucei* and are probably essential for the viability of all trypanosomatids. TbPex3 shows very limited amino acid sequence identity with mammalian Pex3 proteins, including HsPex3, although TbPex3 does contain a Pex19 interaction domain that is conserved in Pex3 proteins of other organisms (Banerjee et al., 2019; Kalel et al., 2019). Given the essentiality of functional glycosomes and the low amino acid sequence identity between TbPex3 and mammalian Pex3 proteins, makes TbPex3 and its interaction with Pex19 an attractive therapeutic target for the treatment of NTDs like African sleeping sickness and American trypanosomiasis.

**FIGURE 1:**
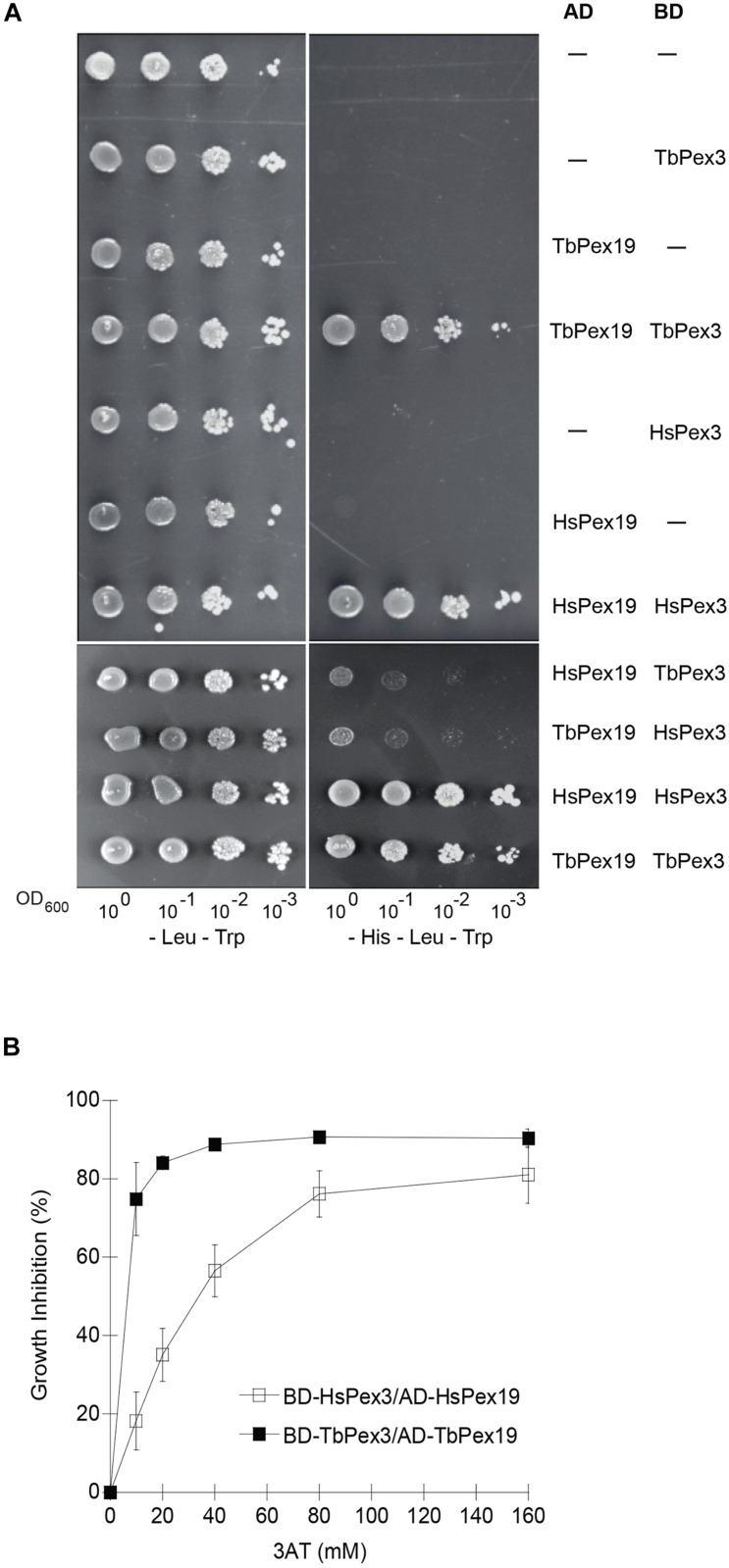
Yeast two-hybrid analysis of protein interactions between TbPex3 and TbPex19, and between HsPex3 and HsPex19. **(A)** *S. cerevisiae* HF7c *pdr5*Δ cells expressing Gal4-AD protein fusions to human (Hs) and *T. brucei* (Tb) Pex19 and Gal4-BD protein fusions to HsPex3 and TbPex3 were grown in liquid synthetic dropout medium and adjusted to an OD_600_ of 1.0. A 10-fold serial dilution series was spotted onto -Leu -Trp (left) and -His -Leu -Trp (right) plates. Growth on -Leu -Trp medium requires cells to have both AD and BD plasmids and is indicative of cell number. Growth on -His -Leu -Trp medium occurs only when there is a protein–protein interaction. **(B)** Sensitivity of HF7c *pdr5*Δ cells expressing *T. brucei* (Tb) or human (Hs) BD-Pex3/AD-Pex19 pairs to 3-aminotriazole (3AT). Basal expression of the *HIS3* gene of yeast is competitively inhibited by 3AT. The BD-TbPex3/AD-TbPex19 interaction showed greater sensitivity than the BD-HsPex3/AD-HsPex19 interaction to the presence of 3AT.

Target-directed drug discovery is often complicated by difficulties in purifying targets or producing them in functional recombinant form, particularly if they are components of multi-protein complexes. Yeast cells expressing foreign proteins have been shown to be useful platforms for screens to identify novel drugs, including anti-parasitics (Blangy et al., 2006; Lentze and Auerbach, 2008; Bilsland et al., 2011; Wong et al., 2017). We adapted the yeast Two-hybrid protein–protein interaction reporter system to screen for compounds that preferentially impair the TbPex3–Pex19 interaction over the HsPex3–Pex19 interaction. Protein–protein interaction in the yeast Two-hybrid system leads to expression of the *HIS3* gene and growth on medium lacking histidine (-His). We used the *Saccharomyces cerevisiae* reporter strain HF7c deleted for the *PDR5* gene encoding the major drug efflux pump (HF7c *pdr5*Δ) for yeast Two-hybrid analysis (Bilsland et al., 2011). As expected, *T. brucei* Pex3 (TbPex3) interacted with TbPex19 and HsPex3 interacted with HsPex19 as shown by growth of strains containing these construct pairs on -His medium (Figure 1A). The lack of yeast growth on -His medium showed that there was no auto activation of any construct. There was marginal growth of strains expressing a human and *T. brucei* Pex protein pair, suggesting that there was limited interaction of Pex3 and Pex19 across species (Figure 1A). Therefore, compounds that disrupt a species-specific Pex3–Pex19 interaction can be found by identifying those compounds that reduce the growth of yeast strains expressing Pex3 and Pex19 from one species.

Basal expression of the *HIS3* gene of yeast is competitively inhibited by 3AT. Before beginning our compound screen using the yeast Two-hybrid system, we performed a titration to define the optimal amount of 3AT to be added to our assay to produce BD-HsPex3/AD-HsPex19 and BD-TbPex3/AD-TbPex19 expressing strains that would be equally sensitive to the addition of compound. We found that the BD-TbPex3/AD-TbPex19 interaction was more sensitive to 3AT with an IC_50_ of 6.2 mM compared to the IC_50_ of 44 mM for the BD-HsPex3/AD-HsPex19 interaction (Figure 1B), suggesting that the strength of interaction between Pex3 and Pex19 in the yeast Two-hybrid system was greater for the human proteins compared to the trypanosomal proteins. Accordingly, 10 mM 3AT was added to the medium for the yeast strain expressing BD-HsPex3/AD-HsPex19 to achieve equal sensitivity with the BD-TbPex3/AD-TbPex19 strain to the addition of compound.

**FIGURE 2:**
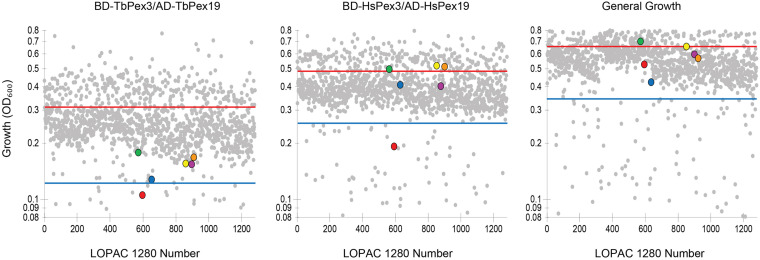
Growth of HF7c *pdr5*Δ cells expressing *T. brucei* (Tb) or human (Hs) BD-Pex3/AD-Pex19 fusion proteins in the presence of compounds from the LOPAC 1280 library. Each compound was at 100 μM final concentration. Each dot represents the level of growth of yeast as measured by OD_600_ in the presence of a particular compound. Cells expressing BD-TbPex3/AD-TbPex19 or BD-HsPex3/AD-HsPex19 were grown in -His -Leu -Trp medium lacking histidine. General growth was measured by growth of HF7c *pdr5*Δ cells containing the parental pGAD424 and pGBT9 two-hybrid vectors in -Leu -Trp medium. The solid red line indicates average level of growth level. The blue line indicates a reduction in the level of growth of 1.5 times the standard deviation. Color legend: 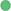 5,7-dichlorokynurenic acid monohydrate, 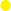 (±)-α-methyl-(4-carboxyphenyl)glycine, 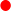 2,3-dimethoxy-1,4-naphthoquinone, 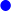 7-cyclopentyl-5-(4-phenoxyphenyl)- 7H-pyrrolo[2,3-d]pyrimidin-4-ylamine, 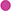 podophyllotoxin, 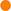 Lidocaine hydrochloride. See Supplementary Tables 1, 2 for details.

**FIGURE 3:**
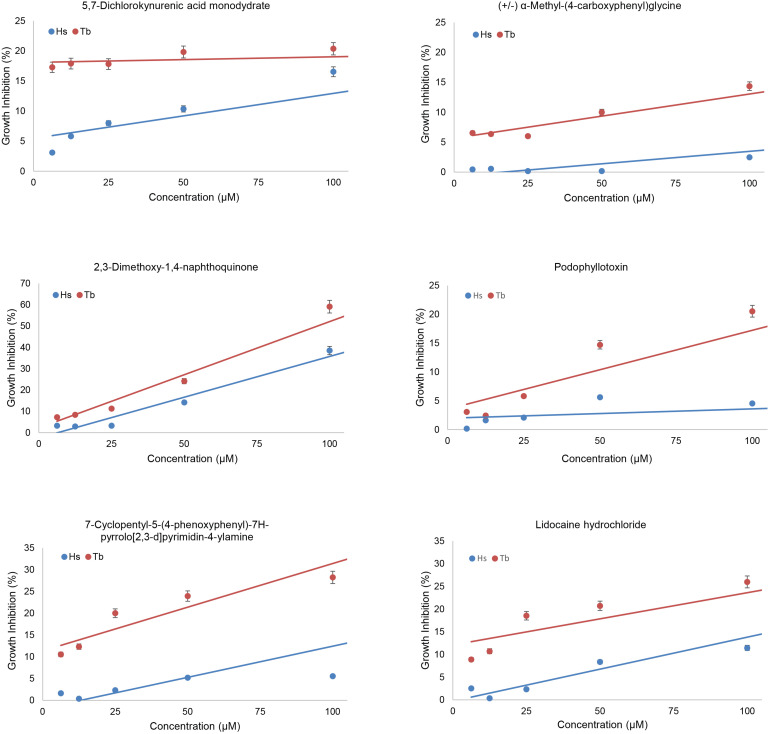
Effects of compounds on growth of yeast two-hybrid strains expressing trypanosomal (Tb) or human (Hs) BD-Pex3/AD-Pex19. The growth of HF7c *pdr5*Δ cells expressing BD-TbPex3/AD-TbPex19 or BD-HsPex3/AD-HsPex19 in the presence of twofold dilutions from 100 to 6.25 μM of the six compounds identified by yeast two-hybrid screening of the LOPAC 1280 library was measured as OD_600_. Growth Inhibition (%) was calculated as 100 – 100 × (growth with compound/growth no compound). The values reported are averages ± SEM of three independent experiments, each done in duplicate.

We screened the LOPAC 1280 to identify compounds that interfered preferentially with the BD-TbPex3/AD-TbPex19 interaction compared to the BD-HsPex3/AD-HsPex19 interaction as measured by inhibition of growth of the respective yeast expression strains in -His -Leu -Trp medium. Non-specific inhibition of growth by compounds was evaluated in -Leu -Trp medium. Each compound was assayed at a final concentration of 100 μM. The extent of inhibition of both BD-Pex3/AD-Pex19 pairs for all compounds tested is presented in Figure 2 and Supplementary Table 1. The compounds assayed are listed in Supplementary Table 2. Six compounds that preferentially inhibited the BD-TbPex3/AD-TbPex19 interaction over its human pairing (Figure 2 and Supplementary Figure 1) were selected for further analysis. Addition of the six compounds at concentrations from 200 to 6.25 μM showed that DMNQ at concentrations up to 200 μM preferentially reduced the survival of yeast harboring the BD-TbPex3/AD-TbPex19 interaction pair over the BD-HsPex3/AD-HsPex19 interaction pair (Figure 3). Accordingly, all subsequent experiments were limited to studies that tested the effects of DMNQ.

### The Small Molecule DMNQ Preferentially Inhibits the Interaction of TbPex3–TbPex19 Over HsPex3–HsPex19

**FIGURE 4:**
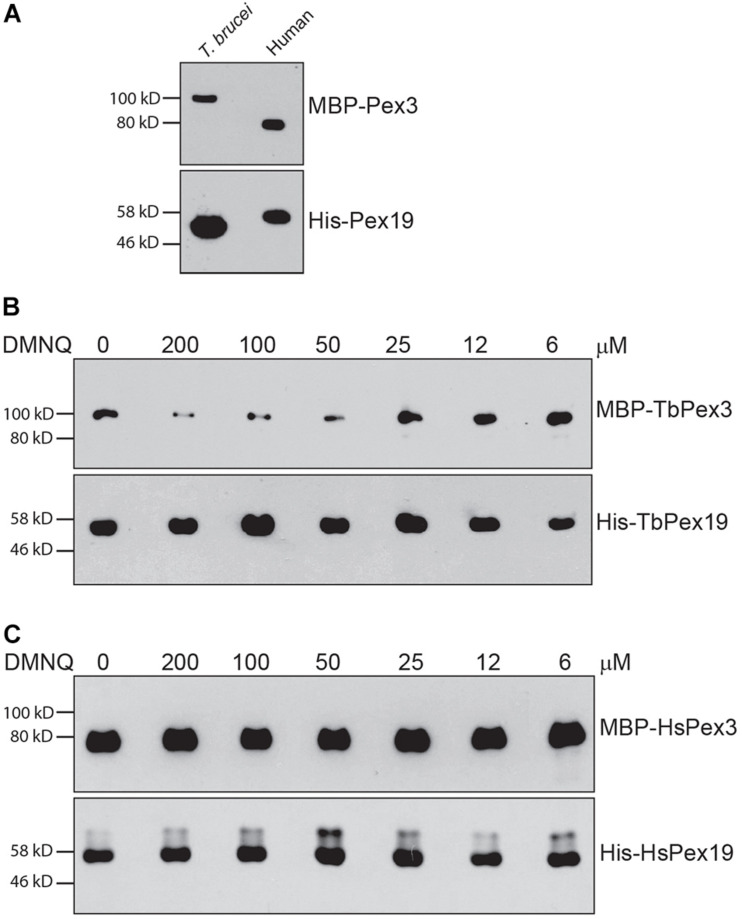
2,3-Dimethoxy-1,4-naphthoquinone preferentially disrupts the interaction of TbPex3/TbPex19 over HsPex3/HsPex19 in a pull-down assay. **(A)** Relative migrations in SDS-PAGE of MBP-fusions to *T. brucei* and human Pex3 proteins and of 6 × His fusions to *T. brucei* and human Pex19 proteins. Fusion proteins were detected by anti-MBP and anti-His-Tag antibodies. **(B,C)** His-Pex19 fusions were immobilized on Ni-Agarose beads and incubated with purified MBP-Pex3 fusion. Bound MBP-Pex3 protein was detected by immunoblotting with anti-MBP antibody. Total His-Pex19 protein was visualized by immunoblotting with anti-His-Tag antibody. Twofold dilutions of DMNQ from 200 to 6 μM were added during incubations. Numbers at left denote migration of molecular mass markers in kilodaltons (kD). Images are representative of three independent experiments.

Trypanosomal Pex3 binds TbPex19 and HsPex3 binds HsPex19 in a pull-down assay (Figure 4A), as previously shown (Sato et al., 2008; Banerjee et al., 2019). Addition of the small molecule DMNQ at increasing twofold concentrations from 6 to 200 μM to the pull-down assay showed that DMNQ reduced the interaction between TbPex3 and TbPex19 but not between HsPex3 and HsPex19 at concentrations between 50 and 100 μM of DMNQ (Figures 4B,C). Off-site effects occurring outside the site of interaction between Pex3 and Pex19 in the yeast cell may underlie the observed growth inhibition of cells expressing BD-HsPex3/AD-HsPex19 by DMNQ at concentrations from 50 to 100 μM (Figure 3). DMNQ and the other five compounds that preferentially inhibited the BD-TbPex3/AD-TbPex19 interaction over its human pairing all showed varying inhibition of yeast general growth (Supplementary Figure 2).

Microscale thermophoresis analysis showed that TbPex3 bound TbPex19 with a *K*_*D*_ of 3.80 ± 1.31 nM (SEM), while HsPex3 bound HsPex19 with a *K*_*D*_ of 4.37 ± 2.30 nM, similar to what has been previously reported (Sato et al., 2008). Titration of DMNQ into the assay increased the apparent *K*_*D*_ of the TbPex3 interaction with TbPex19 by more than 10,000-fold (to 39.6 ± 0.86 μM), while only slightly increasing the *K*_*D*_ of the HsPex3 interaction with HsPex19 (to 14.53 ± 1.97 nM). Therefore, DMNQ preferentially inhibits the binding of TbPex3 to TbPex19 versus the binding of HsPex3 to HsPex19.

### The Small Molecule DMNQ Is Toxic to *T. brucei* Cells Due to Compromised Glycosome Biogenesis

**FIGURE 5:**
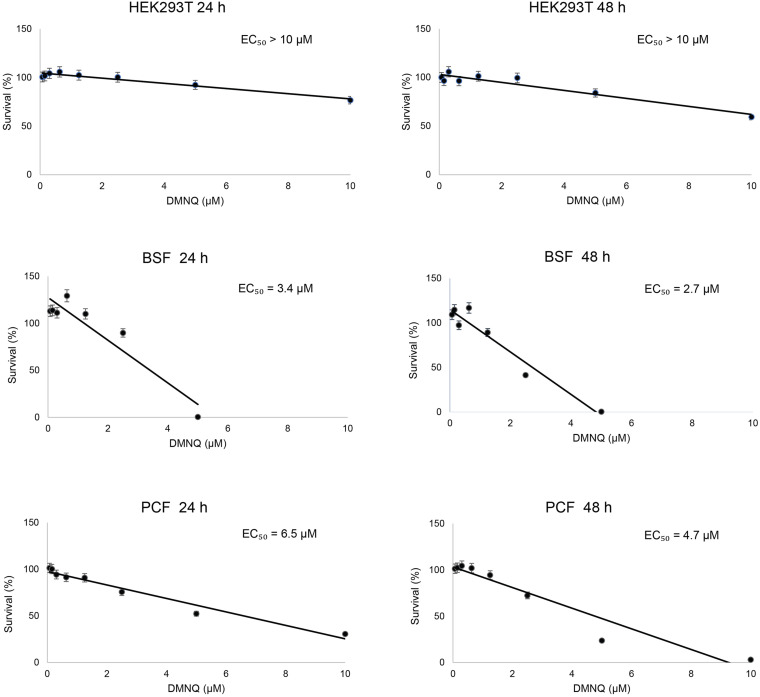
*In vitro* cytotoxicity of DMNQ in HEK293T cells and in *T. brucei* BSF and PCF cells. Cells were incubated with increasing concentrations of DMNQ from 0 to 10 μM for 24 or 48 h. After incubation, cell viability was determined using CellTiter-Glo (CTG). Viability at each concentration of compound is expressed as a percentage of the number of viable cells observed in the absence of DMNQ, which was taken as 100%. A best-fit dose-response curve was generated for each condition and used to determine the half-maximal effective concentration (EC_50_) for each condition. The values reported are averages ± SEM of three independent experiments, each done in duplicate.

We tested the toxicity of the small molecule DMNQ for the BSF and PCF of *T. brucei* and for human HEK293T cells (Figure 5). Twofold dilutions of DMNQ from 0.07 to 10 μM concentration were added to equal numbers of cells for 24 or 48 h and the percentage of surviving cells was determined. The half-maximal effective concentration (EC_50_) for BSF cells was 3.75 and 2.0 μM at 24 and 48 h of treatment with DMNQ, respectively. Low toxicity of HEK293T cells were observed at these concentrations of DMNQ at these respective time points. The EC_50_ for PCF cells was 5.6 and 3.5 μM at 24 and 48 h of treatment with DMNQ, respectively.

**FIGURE 6:**
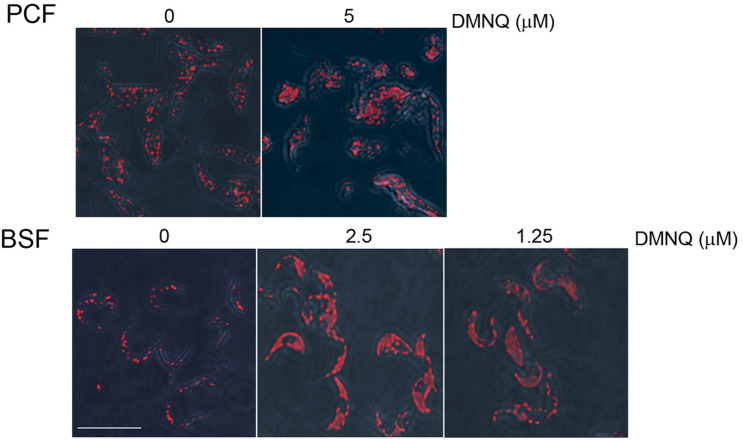
Glycosome biogenesis is disrupted in PCF cells and BSF cells of *T. brucei* by addition of DMNQ. Addition of DMNQ at the concentrations indicated for 48 h led to mislocalization of the glycosomal matrix enzyme aldolase to the cytosol. Aldolase was detected with rabbit anti-aldolase antibodies and Alexa Fluor 568 goat anti-rabbit IgG. Bar, 5 μM.

We performed immunofluorescence analysis on BSF and PCF cells that were untreated or treated with DMNQ for 48 h using antibodies against the glycosomal enzyme aldolase (Figure 6). Aldolase is targeted to the glycosomal matrix by a N-terminal peroxisome targeting signal type 2 (PTS2) (Chudzik et al., 2000). Aldolase showed a predominantly punctate pattern of staining characteristic of glycosomes in untreated BSF and PCF cells. In contrast, BSF and PCF cells treated with DMNQ showed reduced numbers of glycosomes and glycosome biogenesis defects that resulted in a diffuse pattern of staining for aldolase characteristic of mislocalization of the enzyme to the cytosol. A total absence of glycosomes and a total mislocalization of aldolase to the cytosol were not expected, as cells require some fraction of intact functioning glycosomes to remain viable. The compromised glycosome population in DMNQ-treated BSF and PCF cells is consistent with our observations that DMNQ interferes with the interaction between TbPex3 and TbPex19 that is required for the biogenesis of glycosomes (Figure 4).

## Discussion

African sleeping sickness is caused by the protozoan *T. brucei* and, if left untreated, is fatal. Other trypanosomiases inflict additional financial and societal burdens on the peoples of sub-Saharan Africa. Current drugs used to treat trypanosomiases are often limited in their usefulness because of toxicity, complicated administration, side effects, limited efficacy to different stages of disease, and emergent drug resistance. Therefore, the identification of new drug targets and new drugs, as well as the repurposing of old drugs, remains an important venue of investigation for the continued improvement in treatment for trypanosomiases.

Glycosomes are specialized members of the peroxisome family of membrane-enclosed organelles. Glycosomes present an ideal target for drugs that compromise their function or biogenesis because glycosomes are unique to trypanosomatids and house essential metabolic pathways. Like peroxisomes, the biogenesis of glycosomes is dependent on the coordinated activity of proteins called peroxins, or Pex proteins, encoded by the *PEX* genes (Haanstra et al., 2016; Crowe and Morris, 2021). Pex proteins have a diversity of roles in peroxisome biogenesis, including formation of nascent peroxisomes, import of peroxisomal matrix proteins from the cytosol, and control of peroxisomal size and number. The Pex3 protein has been ascribed the role of master regulator of peroxisome biogenesis in all organisms studied. Pex3 functions in making peroxisomes *de novo* from the ER through its interaction with the PMP receptor, Pex19 (Ghaedi et al., 2000; Fang et al., 2004). The identification of a Pex3 protein in trypanosomatids had long remained elusive but was reported independently by two groups in 2019 (Banerjee et al., 2019; Kalel et al., 2019). TbPex3 protein shows limited amino acid sequence identity with Pex3 proteins from other organisms, including human, although TbPex3 like other Pex3 proteins does maintain a Pex19 BD. The essentiality of TbPex3 for glycosome formation and the distinctiveness of its protein primary structure compared to Pex3 proteins from other organisms make TbPex3 an attractive therapeutic target.

Expression of foreign proteins in yeast has been used in the drug discovery process to identify and validate targets and to select affinity reagents for protein targets, such as peptides and small molecules (Blangy et al., 2006; Lentze and Auerbach, 2008; Bilsland et al., 2011; Wong et al., 2017). The yeast two-hybrid system is a widely used genetic assay for the identification and characterization of protein interactions. The yeast two-hybrid system has proved valuable for the screening and characterization of small molecules that inhibit the interactions between medically important proteins (Lentze and Auerbach, 2008; Wong et al., 2017). Given that TbPex3 is distinct in protein primary structure from other Pex3 proteins yet binds its partner protein Pex19 through a domain that is relatively well conserved in all Pex3 proteins makes yeast two-hybrid a system of choice for the screening and identification of small molecules that preferentially inhibit the interaction between TbPex3/Pex19 in comparison to HsPex3/Pex19.

We screened the LOPAC 1280 using the yeast two-hybrid strain HF7c deleted for the *PDR5* gene encoding the major drug efflux pump and identified six small molecules that preferentially inhibited the interaction of TbPex3/TbPex19 over HsPex3/HsPex19. Additional yeast two-hybrid analysis testing the six small molecules individually at different concentrations showed reduced survival of yeast harboring the BD-TbPex3/AD-TbPex19 interaction pair over the BD-HsPex3/AD-HsPex19 interaction pair in the presence of DMNQ. *In vitro* pull-down assays confirmed that DMNQ inhibited the interaction between TbPex3 and TbPex19 while leaving the interaction between HsPex3 and HsPex19 essentially unaffected. The results from the yeast two-hybrid and pull-down assays are consistent with the greatly reduced affinity of TbPex3/TbPex19 (39.6 ± 0.86 μM) compared to HsPex3/HsPex19 (14.53 ± 1.97 nM) for DMNQ, notwithstanding the similar affinities of Pex3 for Pex19 between trypanosomal (*K*_*D*_ = 3.80 ± 1.31 nM) and human (*K*_*D*_ of 4.37 ± 2.30 nM) pairs.

2,3-Dimethoxy-1,4-naphthoquinone was effective at killing both BSF cells and PCF cells, with EC_50_ values ranging from 2.7 to 3.4 μM for BSF cells and 4.7 to 6.5 μM for PCF cells. Small molecule inhibitors of the interaction between trypanosomal Pex5 and Pex14, two components of the glycosomal matrix protein import machinery, showed similar EC_50_ values for *T. brucei* BSF cells (Dawidowski et al., 2017). Human HEK293T cells at these concentrations of DMNQ showed no or limited decrease in viability. Microscopic analysis showed that DMNQ functions in killing both BSF cells and PCF cells of *T. brucei* by interfering with glycosome biogenesis as evidenced by mislocalization of glycosomal matrix enzymes to the cytosol and overall reduced numbers of punctate glycosome profiles, as would be expected by the capacity of DMNQ to interfere with the interaction between TbPex3 and TbPex19. Naphthoquinones have also been reported to cause trypanosomal death by enhanced production of toxic oxygen free radicals (Pieretti et al., 2013) or by targeting parasite enzymes, for example to inhibit specific parasite cysteine proteases (Klein et al., 2020).

**FIGURE 7:**
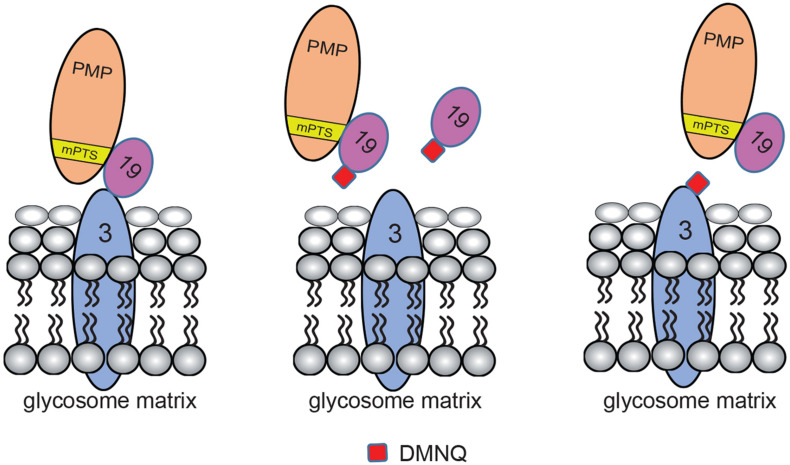
Models for DMNQ docking to TbPex3 and TbPex19. Cartoons showing disruption of the binding between TbPex3 and TbPex19 by DMNQ. The cartoon at extreme left shows TbPex19 with its attached PMP cargo bound to TbPex3. Docking of DMNQ to TbPex19 (middle cartoon) or to TbPex3 (cartoon at extreme right) disrupts the interaction between TbPex3 and TbPex19, leading to failure of import of PMPs and compromised glycosome biogenesis. DMNQ docking to TbPex19 may or may not also impair TbPex19’s binding to its PMP cargo. mPTS, PMP targeting signal.

2,3-Dimethoxy-1,4-naphthoquinone docking to TbPex3 and TbPex19 is modeled in Figure 7. Since DMNQ disrupts the binding between TbPex3 and TbPex19 as shown in pull-down experiments, DMNQ can be envisaged to dock to TbPex3 or TbPex19 either at the site of binding between TbPex3 and TbPex19, or at other sites leading to conformational change in TbPex3 or TbPex19, which in turn disrupts the interaction between TbPex3 and TbPex19. The disrupted interaction between TbPex3 and TbPex19 results in failure to import PMPs, compromised glycosome biogenesis, and death of the *T. brucei* parasite. Whether DMNQ would disrupt the interaction between Pex3 and Pex19 in other *Trypanosoma* spp. or in *Leishmania* spp. remains to be investigated but might be expected given that their Pex3 proteins and TbPex3 exhibit conservation of amino acid sequence of the Pex3 binding interface with Pex19 (Supplementary Figure 3) and their Pex3 proteins do not identify HsPex3 by BLAST analysis but do identify HsPex3 by HHpred analysis, as was reported for TbPex3 (Banerjee et al., 2019).

In closing, we have shown the utility of yeast to screen existent small compound libraries to identify compounds that will selectively kill the zoonotic parasite *T. brucei* by interfering with glycosome biogenesis. Our results identify an expedited approach to identifying other compounds that interfere with glycosome biogenesis or function, thereby expanding the pharmacopeia of compounds that can be used to treat the trypanosomiases.

## Data Availability Statement

The original contributions presented in the study are included in the article/Supplementary Material, further inquiries can be directed to the corresponding author/s.

## Author Contributions

RR conceived the research, analyzed the data, prepared the figures, and co-wrote the manuscript. HB and GE conducted the experiments, analyzed the data, prepared the figures, and co-wrote the manuscript. PL analyzed the data and co-wrote the manuscript. All authors contributed to the article and approved the submitted version.

## Conflict of Interest

The authors declare that the research was conducted in the absence of any commercial or financial relationships that could be construed as a potential conflict of interest.

## Supplementary Material

Supplementary Figure 1 Structures and yeast two-hybrid growth inhibition efficacies of six small molecules of the LOPAC 1280 library selected for further analysis.

Supplementary Figure 2 Effects on yeast growth of the six selected LOPAC 1280 compounds. Compounds are identified by their LOPAC 1280 numbers (see Supplementary Figure 1). General growth was measured using strain H7Fc *pdr5*Δ. Growth Inhibition (%) was calculated as 100 − 100 × (growth with compound/growth no compound). The values reported are averages ± SEM of three independent experiments, each done in duplicate.

Supplementary Figure 3 Alignment of the amino acid sequences corresponding to the binding interface between Pex3 and Pex19 proteins of three *Trypanosoma* spp. and three *Leishmania* spp. Pex3 proteins. Pex3 protein sequences are from *T. brucei* (Tb; Accession no. XP_829090.1), *T. cruzi* (Tc; Accession no. XP_810175.1), *T. vivax* (Tv; Accession no. CCC53628.1), *L. major* (Lm; Accession no. XP_001686984.1), *L. donovani* (Ld; Accession no. XP_003865535.1), and *L. braziliensis* (Lb; Accession no. XP_001569044.1). The number at right denotes the position of the last amino acid in the designated Pex3 sequence. Sequences were aligned using Clustal W (https://embnet.vital-it.ch/ software/ClustalW.html). An amino acid that is identical to its corresponding amino acid in TbPex3 is highlighted in blue. An amino acid that is similar to its corresponding amino acid in TbPex3 is highlighted in yellow. Similar amino acids are grouped as follows: (G, A, S), (A, V), (V, I, L, M), (I, L, M, F, Y, W), (K, R, H), (D, E, Q, N), and (S, T, Q, N). The hydrophobic amino acid of Pex3 required for interaction with Pex19 is designated by an asterisk.

## Abbreviations

BSF: bloodstream form
DMNQ: 2,3-dimethoxy-1,4-naphthoquinone
EC_50_: half-maximal effective concentration
ER: endoplasmic reticulum
NTD: neglected tropical disease
PCF: procyclic form
*PEX*: gene required for peroxisome (glycosome) assembly
Pex: protein encoded by *PEX* gene
PMP: peroxisomal membrane protein.
