# Supplementary figures and images for: A Small Molecule Inhibitor of Pex3–Pex19 Interaction Disrupts Glycosome Biogenesis and Causes Lethality in Trypanosoma brucei
Source: Front Cell Dev Biol. 2021 Jul 19;9:703603. doi: 10.3389/fcell.2021.703603 (PMC8326762; doi:10.3389/fcell.2021.703603)

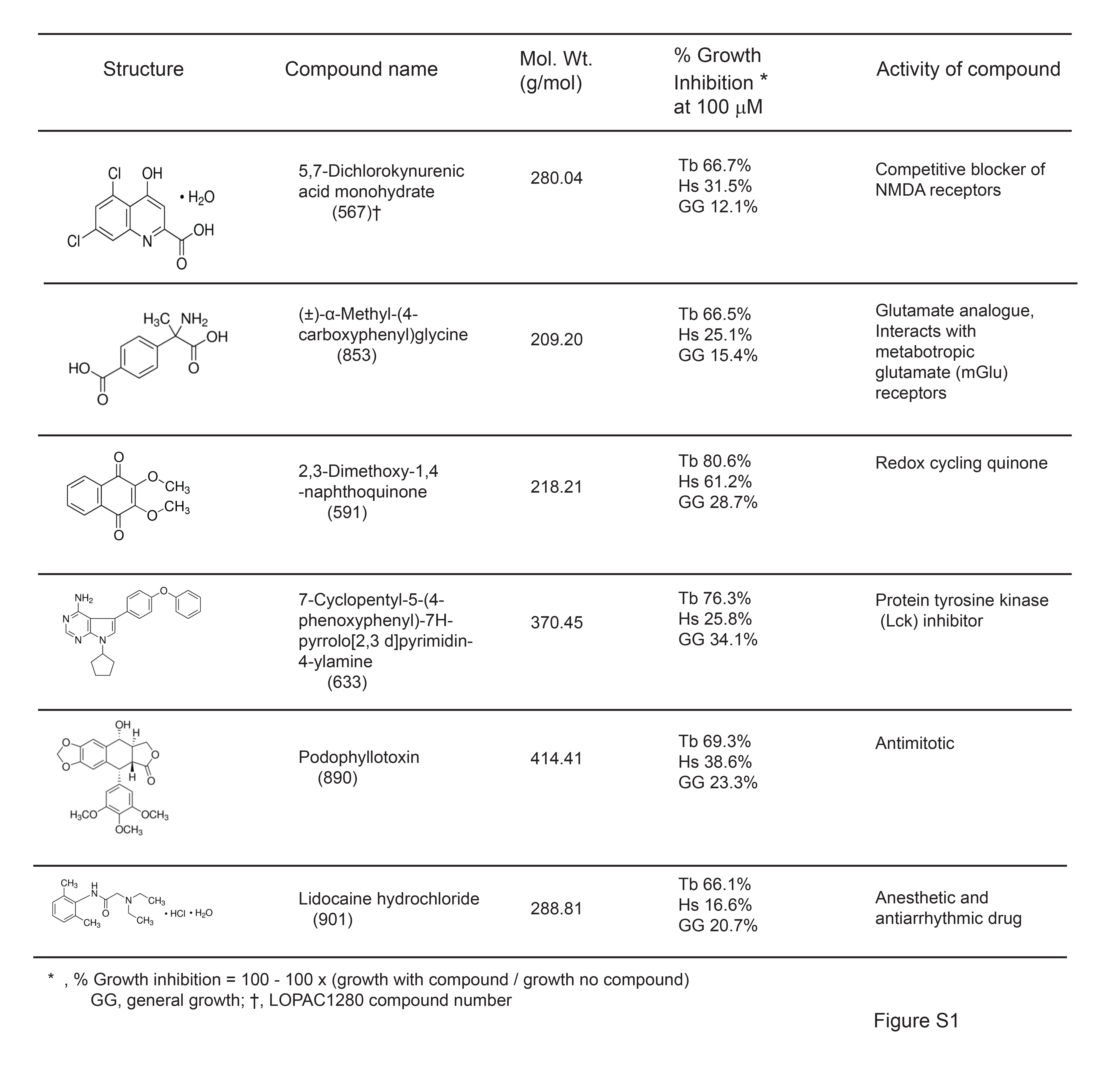

Supplement: Supplementary Figure 1 — Structures and yeast two-hybrid growth inhibition efficacies of six small molecules of the LOPAC 1280 library selected for further analysis. [file Image_1.jpg]

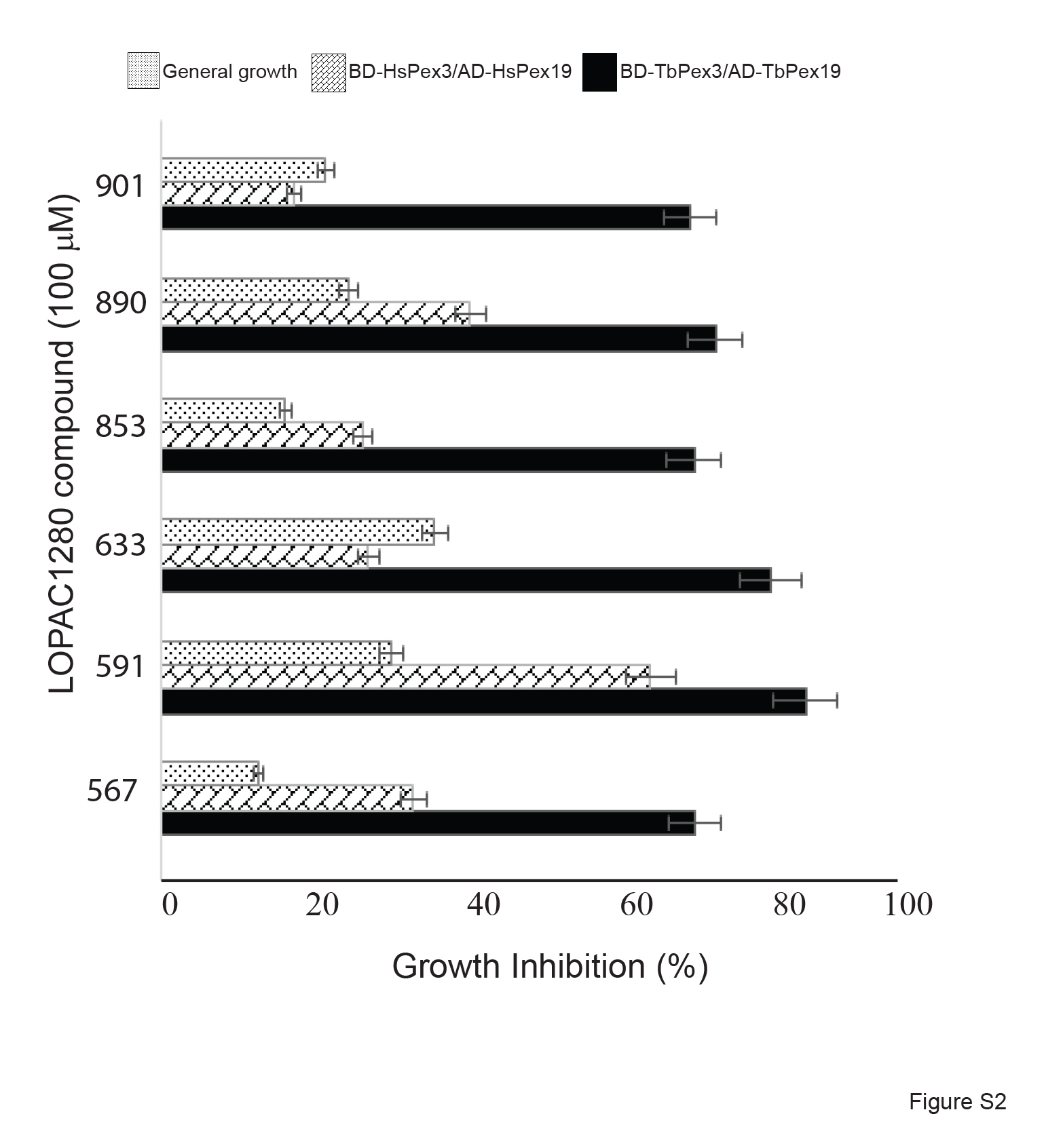

Supplement: Supplementary Figure 2 — Effects on yeast growth of the six selected LOPAC 1280 compounds. Compounds are identified by their LOPAC 1280 numbers (see Supplementary Figure 1). General growth was measured using strain H7Fc pdr5Δ. Growth Inhibition (%) was calculated as 100 − 100 × (growth with compound/growth no compound). The values reported are averages ± SEM of three independent experiments, each done in duplicate. [file Image_2.JPEG]

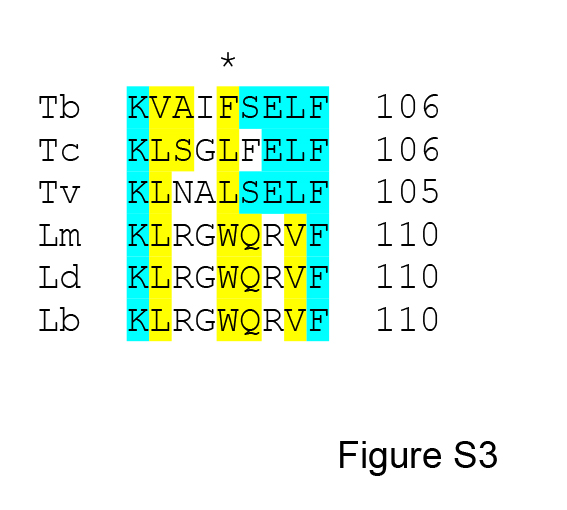

Supplement: Supplementary Figure 3 — Alignment of the amino acid sequences corresponding to the binding interface between Pex3 and Pex19 proteins of three Trypanosoma spp. and three Leishmania spp. Pex3 proteins. Pex3 protein sequences are from T. brucei (Tb; Accession no. XP_829090.1), T. cruzi (Tc; Accession no. XP_810175.1), T. vivax (Tv; Accession no. CCC53628.1), L. major (Lm; Accession no. XP_001686984.1), L. donovani (Ld; Accession no. XP_003865535.1), and L. braziliensis (Lb; Accession no. XP_001569044.1). The number at right denotes the position of the last amino acid in the designated Pex3 sequence. Sequences were aligned using Clustal W (https://embnet.vital-it.ch/ software/ClustalW.html). An amino acid that is identical to its corresponding amino acid in TbPex3 is highlighted in blue. An amino acid that is similar to its corresponding amino acid in TbPex3 is highlighted in yellow. Similar amino acids are grouped as follows: (G, A, S), (A, V), (V, I, L, M), (I, L, M, F, Y, W), (K, R, H), (D, E, Q, N), and (S, T, Q, N). The hydrophobic amino acid of Pex3 required for interaction with Pex19 is designated by an asterisk. [file Image_3.JPEG]
